# n-Type redox-tuneable conducting polymer optical nanoantennas†

**DOI:** 10.1039/d4tc03038a

**Published:** 2024-10-02

**Authors:** Suraya Kazi, Pravallika Bandaru, Haoran Tang, Yulong Duan, Shangzhi Chen, Fei Huang, Magnus P. Jonsson

**Affiliations:** a Organic Photonics and Nanooptics group, Laboratory of Organic Electronics, Department of Science and Technology, Linköping University, Campus Norrköping SE 601 74 Sweden magnus.jonsson@liu.se; b Institute of Polymer Optoelectronic Materials and Devices, State Key Laboratory of Luminescent Materials and Devices, Guangdong Basic Research Center of Excellence for Energy & Information Polymer Materials, South China University of Technology Guangzhou China

## Abstract

Conducting polymers can be dynamically switched between being optically metallic (negative real permittivity) and dielectric (positive real permittivity) by varying their redox state. This has enabled nanoantennas with plasmonic resonances that can be reversibly turned on/off, opening for applications in dynamic metaoptics, reflective displays, and smart windows. However, previous reports on conducting polymer plasmonics were limited to p-type polymers. Here, we show that a highly conducting n-type polymer, called poly(benzodifurandione) (PBFDO), can also provide optically metallic properties and be used to make dynamic optical nanoantennas. The doped version of the polymer becomes metallic at wavelengths above around 700 nm, leading to plasmonic extinction peaks for nanodisks made from the material. These peaks can be reversibly switched off and on electrically or chemically by varying the doping level of the polymer. The study extends the field of dynamic polymer plasmonics to n-type materials and broadens the application areas of PBFDO.

## Abstract 2

We show that the n-type conducting polymer PBFDO can be optically metallic for wavelengths above around 695 nm after post-treatment, and that nanostructures of the material can act as optical nanoantennas that are dynamically switchable.

## Graphical abstract

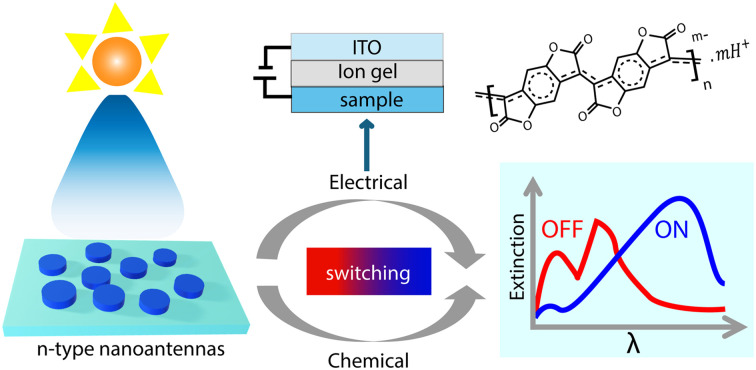

## Introduction

The field of plasmonics was recently extended to organic conducting polymers.^1,2^ In contrast to metals like gold or silver, the plasmonic properties of conducting polymers can be reversibly tuned or completely switched off and on repeatedly by modulating their doping level, spurring intense research on dynamic polymer plasmonics and metasurfaces.^2^ Examples of demonstrations include switchable diffraction and flat lenses.^3,4^ In addition, conducting polymers can provide unusual features such as in-plane hyperbolic properties by aligning the polymer chains.^5^ The research area of conducting polymer plasmonics is in its early phase and has so far been limited to a few p-type materials,^1,6^ mostly focusing on different types of PEDOT (poly[3,4-ethylenedioxythiophene]). Here, we report for the first time redox-tunable plasmonic properties of nanoantennas made from an n-type conducting polymer. The polymer of choice is called poly(benzodifurandione) (PBFDO), which was recently reported as a highly conducting n-type polymer with excellent ambient stability due to exceptionally low value of the lowest unoccupied molecular orbital (LUMO) level.^7,8^ This new addition to n-type organic materials constituted a breakthrough in the field of organic electronics, providing conductivity on par with the typical best p-type counterparts. PBFDO (also called n-PBDF) has already been used for organic electrochemical transistors^7,9^ thermoelectrics,^7^ and electrochromic displays.^8^ However, the capability of PBFDO for nanooptics and plasmonics has not yet been explored, which forms the topic of this study. Based on ellipsometry measurements, we show that the material can possess optically metallic properties in its doped state, shown through negative values of its real permittivity (*ε*_1_ < 0). This metallic spectral range started at the upper end of the visible, with the transition point from positive to negative permittivity at around 700 nm. We then show that PBFDO could switch to a non-metallic state by dedoping, for which the real permittivity was instead positive in the whole measured spectral range. The material thereby offers reversible switching between metallic and dielectric properties, showing promise for dynamic polymer plasmonic metasurfaces as previously only shown for p-type polymers.^1,6^ To examine this possibility, we structured the material into non-periodic arrays of nanodisks. The results show clear extinction peaks with positions that vary with nanodisk size as expected from plasmonic resonances and in agreement with complementary simulations. We further show that the plasmonic resonance peaks could be modulated by varying the doping level of the material, both by chemical means and by electrical potentials. The study extends the topic of dynamic conducting polymer plasmonics to n-type materials, opening up various types of tunable metasurfaces and other nanooptical devices with novel functionalities.

## Results and discussion

**Fig. 1:**
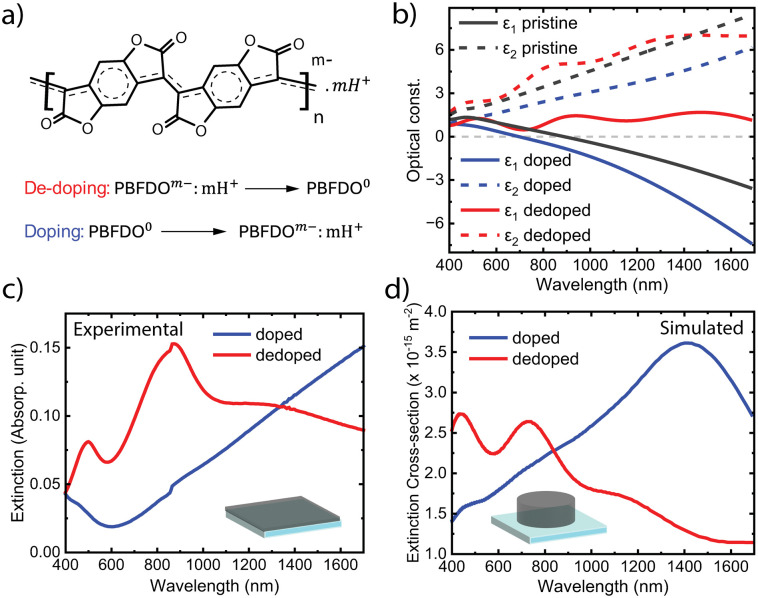
(a) Chemical structure of n-doped PBFDO, and its doping/dedoping behaviour. (b) In-plane permittivity dispersion for PBFDO thin films in the pristine state (black), after doping (blue) and de-doping (red). Full lines correspond to the real component (*ε*_1_) and dashed lines correspond to the imaginary component (*ε*_2_). (c) Experimental extinction spectra of a PBFDO thin film (40 nm thickness) after chemical doping (blue) and dedoping (red). (d) Simulated extinction cross-section of a single PBFDO nanodisk on glass in its doped (blue) and dedoped (red) states (80 nm diameter, 30 nm height).

Fig. 1a presents the molecular structure of PBFDO and its reversible doping behaviour. By contrast to many other conducting polymers, the counterions to maintain charge balance upon doping can be simply protons. We prepared thin films of PBFDO by spin-coating PBFDO solution in dimethyl sulfoxide (DMSO) onto glass substrates followed by annealing inside a N_2_-regulated glovebox. The films were then further treated by exposing them to the vapour of a reductant called branched polyethyleneimine (PEI). For dedoping, we soaked the films in sulphuric acid solution, which neutralizes the negative charge carriers by oxidation. More details can be found in the Experimental section.

Fig. 1b shows the permittivity of PBFDO films in the pristine state (black), after PEI treatment (blue) and after dedoping (red). The permittivity dispersions were retrieved by ellipsometry and fitting to a Drude–Lorentz model.^10^ We find that the doped pristine film shows optically metallic behaviour for wavelengths above around 890 nm (at the plasma frequency), where it transitions from positive to negative real permittivity (*ε*_1_ < 0). In turn, negative *ε*_1_ shows promise for the use of PBFDO for plasmonics. The transition wavelength to metallic behaviour could be further blueshifted to around 700 nm by PEI vapor treatment, which also significantly reduced the imaginary permittivity (*ε*_2_) corresponding to optical losses. This is particularly interesting considering that previous work indicated that the alkaline medium can cause ring opening side reaction of lactones.^11,12^ Conductivity measurements confirmed that the PEI-treated PBFDO films maintained high electrical conductivity, although often with decreased values from those of the pristine material (Table S1 in the ESI†). The combination of blueshifted plasma frequency without improved or even lowered electrical conductivity could be related to several things, such as increased carrier density with lowered mobility and/or differences in the contributions to the permittivity dispersions from the neutral state and polaron absorption features.^5^ In terms of optically metallic properties, the PEI vapour-treated PBFDO rivals that of the best p-type conducting polymers (see Table S2 in the ESI†). Indeed, PEDOT:Sulf showed plasma frequency that was also around 700 nm.^1^ Commonly used PEDOT:PSS typically becomes metallic at longer wavelengths due to lower charge carrier density (and/or larger carrier mass), with the epsilon-near-zero position ranging from over 5000 nm to 865 nm depending on post-treatments.^13^ Dedoping PBFDO films by exposure to sulphuric acid completely changed the permittivity of the material. The dedoped PBFDO showed positive real permittivity (*ε*_1_ > 0) throughout the measured spectral range. The non-monotonic features in the dedoped state relate to the neutral optical gap and polaron transitions of the neutral polymer, as also present for *ε*_2_. For the whole measured spectral range, *ε*_2_ was larger in the dedoped state than in the doped state.

The permittivity results agree well with extinction spectra of PBFDO thin films in their doped (which refers to the PEI treated material from now on) and dedoped states (around 30 nm thickness, Fig. 1c). In the doped state, the material shows low extinction in the visible region and a gradual increase towards longer wavelengths due to charge carrier absorption and reflection by the conducting material. By contrast, an energy gap and polaron absorption peaks emerge for the dedoped film, in agreement with the permittivity dispersion and previous works.^8^

The possibility to switch PBFDO between optically metallic and dielectric states holds promise for using the material to create dynamic plasmonic nanoantennas. Fig. 1d presents the simulated optical response for a PBFDO nanodisk of 80 nm diameter and 30 nm height in its two states (using the measured permittivity in Fig. 1b). The doped nanostructure possesses a clear extinction peak at around 1400 nm. This response is distinctly different from the extinction of the (non-structured) doped PBFDO, which lacks features in this range and only shows a monotonic increase in extinction with wavelength. Just as for conventional plasmonic metals, the extinction peak instead stems from the nanostructuring of the optically metallic material. This peak was not present in the extinction spectrum of the dedoped nanodisk, which is no longer metallic. The extinction of the dedoped nanodisk was instead similar to that of the non-structured dedoped film, primarily showing the neutral optical gap and polaron peaks.^8^

**Fig. 2:**
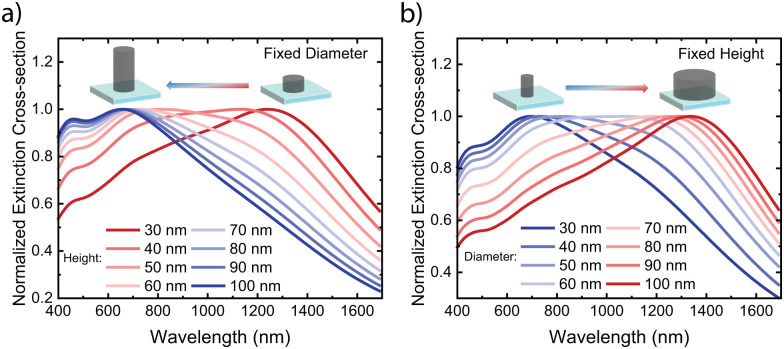
Simulated normalized extinction cross-section of a single nanodisk of PBFDO on glass having: (a) fixed diameter (50 nm) and varying height (from 30 nm to 100 nm with 10 nm interval), (b) fixed height (50 nm) at varying diameter (from 30 nm to 100 nm with 10 nm interval).

To understand the mechanism behind the extinction peak of doped PBFDO nanodisks, we explored the dependence on nanodisk geometry. Fig. 2a (and Fig. S1a, ESI†) shows that the extinction peak position shifts towards shorter wavelengths as the nanodisk height increases while keeping the diameter fixed at 50 nm. Conversely, for nanodisks with a fixed height of 50 nm, the peak position shifts towards longer wavelength with increasing diameter (Fig. 2b and Fig. S1b, ESI†). These results, exhibiting a blue-shift with increasing height and a red-shift with increasing diameter of the nanodisk, agree well with the geometry-dependence behaviour observed in plasmonic nanodisk antennas made from conventional metals^14^ as well as p-type conducting and semiconducting polymers.^1,6^ The result can be understood based on the dependence on aspect ratio (diameter/height) on the polarizability for a plasmonic oblate spheroid.^1,14^

**Fig. 3:**
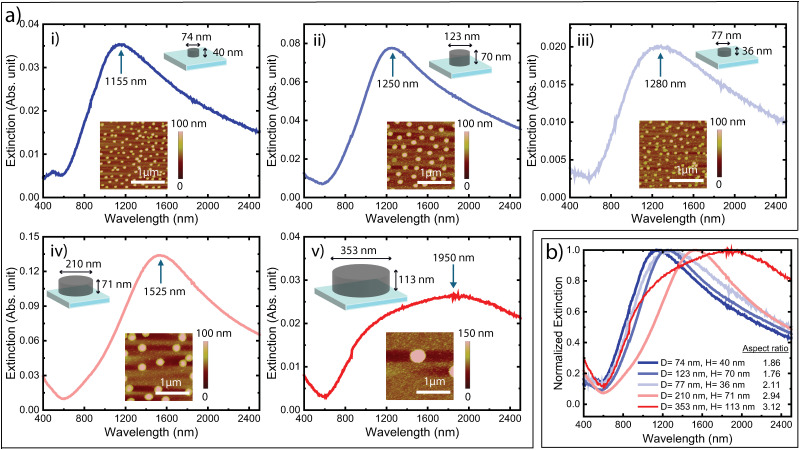
(a) Experimental optical extinction spectra of samples coated with PBFDO nanodisks of different dimensions: (i) *D* = 74 ± 4 nm, *H* = 40 ± 8 nm, *P* = 161 nm; (ii) *D* = 123 ± 6 nm, *H* = 70 ± 6 nm, *P* = 309 nm; (iii) *D* = 77 ± 10 nm, *H* = 36 ± 3 nm, *P* = 198 nm; (iv) *D* = 210 ± 10 nm, *H* = 71 ± 8 nm, *P* = 442 nm; (v) *D* = 353 ± 19 nm, *H* = 113 ± 14 nm, *P* = 2073 nm, where *D*, *H* and *P* denote the average diameter, average height and average centre-to-centre distance of the nanodisks, respectively. Insets show atomic force microscopy (AFM) images of the samples and the schematics with size indications. (b) Normalized extinction spectra of the nanodisk samples presented in panel a (i–v).

For experimental verification, we used colloidal lithography to produce samples coated with non-periodic arrays of PBFDO nanodisks.^1,15^ As detailed in the Methods section, we self-assembled polystyrene nanospheres on PBFDO thin films on glass substrates and used reactive ion etching (RIE) to remove the polymer material between but not under the nanospheres. After removal of the nanospheres, the final samples contain PBFDO nanodisks arranged in a non-periodic manner on the substrates. Fig. 3a presents extinction spectra of samples with different nanodisk diameters and heights, as controlled by the nanosphere size and film thickness, respectively. The insets present atomic force microscopy (AFM) images of the corresponding samples, and the schematics indicate the measured dimensions of the nanodisks. We first note that the experiments verify the simulated predictions in terms of presenting clear optical extinction peaks for all different nanodisk sizes. We stress again that such peaks are not present for the non-structured doped PBFDO films. The lower extinction value of the sample with the largest nanodisks (in Fig. 3a–v) is attributed to a sparser distribution of nanodisks, which can be seen in AFM images with larger scan size (Fig. S2, ESI†). The normalized extinction spectra of these nanodisks, presented in Fig. 3b, further show that the peak position generally redshifted with increasing aspect ratio (diameter/height) of the nanodisks, also in accordance with the simulations and with the behaviour of plasmonic nanodisks of PEDOT:Sulf^1^ and traditional metals.^14^ In more detail, samples with aspect ratios of 1.86, 1.76, 2.11, 2.94, and 3.12 possessed extinction peaks at 1155 nm, 1250 nm, 1285 nm, 1525 nm, and 1950 nm, respectively.

**Fig. 4:**
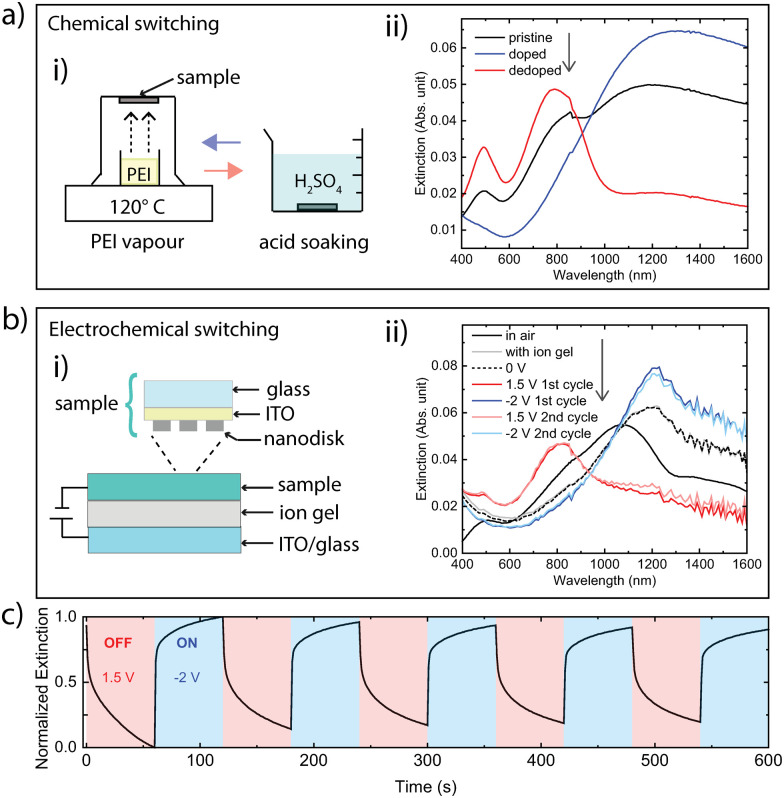
Dynamic tuning of PBFDO optical nanoantennas: (a) chemical switching of nanodisks on glass (diameter = 131 ± 8 nm, height = 35 ± 7 nm), with a schematic of the tuning principle in (i) and extinction spectra in (ii). (b) Electrical switching of PBFDO nanodisks on an ITO/glass substrate (diameter = 149 ± 9 nm, height = 44 ± 8 nm) with a schematic of the device and tuning principle in (i) and extinction spectra in (ii). (c) Normalized extinction of PBFDO nanoantennas (same sample as in panel b-ii) at its plasmonic peak position (at 1215 nm) over time during five cycles of electrical switching between 1.5 V and −2 V with a duration of 1 min for each step. The arrows in panels (a-ii) and (b-ii) indicate the sequence of the obtained spectra.

We turn to the important possibility to reversibly turning the nanoantennas on/off *via* the polymer redox state, which switches the material between metallic and dielectric optical behaviour. Fig. 4a presents the results for chemical tuning for a sample with 131 nm diameter nanodisks with 35 nm height (see AFM image in Fig. S3, ESI†). The schematic to the left depicts the chemical tuning procedure. The extinction for the pristine state (directly after nanofabrication, black curve) shows clear absorption features of the undoped PBFDO as well as a peak with moderate magnitude at longer wavelengths (around 1200 nm). In accordance with the permittivity results, PEI vapour treatment suppressed neutral state absorption and improved the optically metallic behaviour of the nanodisks in terms of providing a more distinct nanoantenna peak, at around 1300 nm. The nanoantennas could then be turned off again by dedoping the material by acid treatment, leading to disappearance of the antenna peak and reemergence of the neutral state features of the polymer. Simulations confirm this behaviour (Fig. S4, ESI†).

To also demonstrate electrical switching, we produced PBFDO nanodisks (149 nm diameter, 44 nm height, see AFM image in Fig. S5, ESI†) on substrates coated with indium tin oxide (ITO). Electrical switching was then enabled by coating the sample with an ionic gel-like electrolyte and finalizing the circuit using another ITO/glass substrate (see schematic in Fig. 4b-i). The ITO-coated substrate and the ion gel were transparent in the relevant spectral range and without any prominent features (Fig. S6, ESI†). Fig. 4b-ii presents extinction spectra at different bias potentials, as well as for the pristine sample in air and after device assembly. The pristine sample in air presented a clear peak at around 1070 nm. The position of this peak redshifted upon device assembly to around 1215 nm, which is in accordance with shifts in plasmonic resonances due to an increased refractive index of the surrounding medium.^16,17^ Connecting the device at 0 V did not cause observable changes to the extinction spectrum. However, further doping the material by applying a negative bias (−2 V) increased the intensity of the extinction peak. More drastic changes were induced by applying a positive bias (1.5 V), which fully suppressed the long-wavelength nanoantenna peak and the sample only presented the optical behaviour of the dedoped material. The nanodisk device could then be repeatedly switched between doped (ON) and dedoped (OFF) states by alternating the bias potential between – 2 V or +1.5 V. The reversible nature of the electrochemical switching is also shown in Fig. 3c, which presents the variation in extinction over time at the nanoantenna peak (1215 nm) for multiple cycles. The results show that the process of electrochemical doping from the dedoped state (from 1.5 V to −2 V) took around 1 s to reach 67% (or 5 s to reach 80%) of the maximum value of the normalized extinction. Dedoping (from −2 V to 1.5 V) was slower, for which the normalized extinction dropped to around 72% of the maximum value after 1 s (or to 51% after 5 s). Fig. S7 (ESI†) presents a cyclic voltammogram (CV) of the switching process.

## Conclusions

Our study extends conducting polymer plasmonics and nanooptics to n-type polymer materials. This was enabled thanks to the high charge density and conductivity of n-doped PBFDO, which can provide optically metallic properties for wavelengths above 700 nm. Owing to the possibility to vary the charge density *via* the redox state of the polymer, PBFDO nanoantennas can be dynamically turned off and on again by chemical or electrical stimuli. Future studies may use this principle to create dynamically functional metasurfaces including flat ultrathin lenses and holograms. This work also opens up the potential for dynamic systems based on combinations of n- and p-type plasmonic polymers.

## Experimental

### Sample preparation

We cleaned glass substrates (25 × 25 × 1 mm^3^) with four steps of ultrasonication by submerging the substrates first in soap solution (with Hellmanex in deionized (DI) water with a ratio of 1 : 10) followed by DI water, acetone and finally iso-propanol with each step for 10 minutes. The substrates were dried using a nitrogen gun. The thin film deposition of PBFDO was conducted inside a glovebox. PBFDO solution in DMSO (bought from Volt-Amp Optoelectronics Tech. Co.) with a concentration of 10 mg mL^−1^ was first stirred rigorously at 50 °C for 1–2 h to ensure uniformity of the solution. Detailed characterization of the material can be found in ref. 7. Clean substrates were treated with UV-ozone for 15 minutes. The substrates were pre-heated at 110 °C and immediately spin-coated with PBFDO solution at 1000 rpm for 1 minute inside a N_2_-containing glovebox. After spin-coating, the samples were annealed at 110 °C for 10 minutes to remove excess DMSO.

### Nanostructure fabrication

Nanostructures of PBFDO were created by colloidal lithography. First, 2% solution of poly(diallyldimethylammonium chloride) (PDDA), a cationic polyelectrolyte, in water was drop-casted on PBFDO film in order to create a positively charged surface on the PBFDO thin film. After 2 minutes, the sample was rinsed with DI water for 30 s and dried with N_2_. 0.1% solution of polystyrene (PS) nanoparticles in water was drop-casted on the sample and left to sit for 5–10 minutes. The positively charged surface attracts a certain amount of negatively charged PS beads and excess PS beads are removed by rinsing with DI water for 30 s. The samples were then etched by RIE (Advanced Vacuum-Vacutec) for 20–140 s with 50 W power, 200 mTorr pressure and 400 sccm oxygen flow. PS nanoparticles worked as the masks during etching, which left the PBFDO nanodisks underneath. PS beads were later removed using tape.

### Chemical doping–dedoping process

The doping level of pristine or dedoped PBFDO films/nanostructures could be enhanced using branched PEI (*M*_w_ ∼ 800, Sigma-Aldrich) as a reductant. PEI was heated at 130 °C and the samples were exposed to its vapour for 10 minutes followed by 10 min of annealing inside an N_2_-regulated glovebox. The samples were then rinsed with water and iso-propanol and dried with N_2_ in order to remove excess PEI from the sample surface. For dedoping, the samples were soaked in 0.1 M H_2_SO_4_ solution for 30 min, dried with N_2_ and left in air to oxidize further by reacting with the oxygen in air. The use of higher concentrations (1 M H_2_SO_4_) could dedope the samples faster and provide prominent dedoped absorption features but could also damage the samples.

### Material characterization

The permittivity of the PBFDO thin films at doped and dedoped states was obtained from ellipsometry measurements performed using a RC2 Mueller matrix ellipsometer (J. A. Woollam Co., Inc). Samples were made onto 430 μm thick single-side-polished c-plane sapphire wafers (Shenyang Ebetter Optics Co., Ltd). Ellipsometry data were collected under normal ambient conditions at four incident angles (45°, 55°, 65° and 75°) within a spectral range from 210 nm to 1690 nm and were analyzed in CompleteEase software (J. A. Woollam Co.) using an isotropic Drude–Lorentz model. Check our previous works in ref. 1 and 10 for more details.

### Morphological characterization

The thickness of PBFDO thin films was determined by a Dektak profilometer. To obtain the geometry of the nanostructures, the sample surface was scanned at 2.54 Hz in tapping mode on an AFM device (Veeco Dimension 3100). AFM images were analyzed using Nanoscope Analysis software (Bruker) to determine the height profile and ImageJ2 Fiji software for the diameter of the nanodisks by taking an average of the dimensions of 10 nanodisks on the sample.

### Numerical optical modelling

Finite difference time domain (FDTD) commercial software Ansys Lumerical FDTD (https://www.ansys.com/products/optics/fdtd) was used to simulate the optical response of PBFDO nanoantennas. In the FDTD simulation region, a PBFDO nanodisk was placed on a SiO_2_ substrate (refractive index 1.5), and the superstrate was used as air (refractive index 1). The dielectric permittivity of PBFDO used in the simulations was obtained from ellipsometry measurements. Perfectly matched layers were used in the *X*, *Y*, and *Z* directions. The PBFDO antenna was illuminated with a total field/scattered field source at normal incidence in the *Z*-direction. Cross section monitors were used to calculate scattering and absorption cross sections of the nanoantenna.

### Electrochemical switching

For switching the nanoantennas electrically, we fabricated them on ITO-coated glass substrates (pre-cut with dimensions of 2.5 × 2.5 × 0.7 cm^3^ with 50 nm of ITO layer, 30–40 Ω sq^−1^ resistance) following the same procedure as mentioned above for the nanoantennas on glass. A blank ITO substrate was used as the counter electrode and an ion gel was used as the electrolyte. To prepare the ion gel solution, we mixed a copolymer poly[(vinylidene fluoride)-*co*-hexafluoropropylene] (PVDF–HFP, *M*_n_ = 130 000 from Sigma-Aldrich) in acetone with a weight ratio of 1 : 7 and stirred overnight at 50 °C. Later 1-ethyl-3-methylimidazolium bis(trifluoromethylsulfonyl)imide (EMIM TFSI, ≥97.0% from Sigma-Aldrich) was added to the PVDF–HFP solution in acetone with a weight ratio of 1 : 2 and stirred at 50 °C for 40 min. The solution was then drop-casted with a thin layer on clean glass slides and heated at 60 °C for 2 h in order to remove acetone from the gel. More details about the process of ion gel preparation can be found in ref. 18. The well-dried ion gel was later transferred onto the sample carefully. A clean ITO/glass substrate (counter electrode) was placed on the ion gel making sure that the ITO layer was in contact with the ion gel. To ensure proper contact of the sample with the electrolyte, the sample and the blank ITO/glass substrate, having ion gel in the middle, were attached together on the edge with tape. The sample and the blank ITO/glass substrate were then connected to a compact potentiostat/galvanostat (Metrohm Autolab PGSTAT204), combined with NOVA software, and required voltage biases were applied in order to electrochemically switch the nanoantennas. The electrochemical setup was placed inside the spectrometer in order to simultaneously measure the spectra during switching.

### Optical characterization

A UV-vis-NIR spectrometer (PerkinElmar Lambda 900) was used to measure the extinction (combining absorption and scattering) spectra of the samples within the wavelength range of 400 nm to 2500 nm with an interval of 5–10 nm. For measuring the change in extinction at the plasmonic peak position during multiple cycles of electrical switching, time-drive mode was used.

## Author contributions

S. C and M. P. J conceived the project and designed the study together with S. K. PBFDO was synthesized by H. T. and F. H. Experiments and characterizations were performed by S. K. P. B performed the optical simulations. M. P. J and S. C supervised the project. The project and results were regularly discussed among M. P. J, S. C., S. K., Y. D., and P. B. M. P. J and S. K wrote the manuscript with input from the other authors.

## Data availability

The data are available *via* Zenodo at https://doi.org/10.5281/zenodo.13771432.

## Conflicts of interest

The authors declare no conflict of interest.

## Supplementary Material

TC-012-D4TC03038A-s001
