# Supplementary material for: n-Type redox-tuneable conducting polymer optical nanoantennas
Source: J Mater Chem C Mater. 2024 Oct 2;12(43):17469–74. doi: 10.1039/d4tc03038a (PMC11445732; doi:10.1039/d4tc03038a)
Supplement: TC-012-D4TC03038A-s001 [file TC-012-D4TC03038A-s001.pdf]

Supplementary Information (SI) for Journal of Materials Chemistry C.
This journal is © The Royal Society of Chemistry 2024

Supporting Information for

n-Type redox-tuneable conducting polymer optical nanoantennas

Suraya Kazi,[a] Pravallika Bandaru,[a] Haoran Tang,[b] Yulong Duan,[a] Shangzhi Chen,[a] Fei Huang,[b] and Magnus P. Jonsson*[a]

Table S1: Electrical conductivity of PBFDO thin films in their pristine state vs after being treated with PEI and rinsed with IPA and water. Samples were prepared by depositing gold lines on glass substrates and spincoating PBFDO thin film on top. Thickness measurement was done using a Dektak profilometer and the resistance was measured by 4-line probe using a field effect transistor (FET) station with a potentiostats (Keithley 4200-SCS).

Sample #	Conductivity (S/cm)	
	pristine	PEI treated and rinsed
1	1474.1 ± 236.3	797.8 ± 127.9
2	875.5 ± 107.4	703.6 ± 86.3
3	640.5 ± 78.6	623.6 ± 76.5

Table S2: Comparison of different properties of PBFDO to other conducting polymers.

Type	Coducting polymer	ENZ wavelength, nm	LUMO level, eV	Air stability
P-type	PEDOT:PSS	5267[1]	-	stable
	Acid-treated PEDOT:PSS	865[1]	-	stable
	PEDOT:PSS	1300[2]	-	stable
	PEDOT:Tos	1177[3]	-	stable
	PEDOT:Sulf	700[4]	-	stable
n-type	n-doped PBFDO	890*	-5.1[5]	stable
	PEI-treated PBFDO	695*	-	stable
	N2200	-	-3.90[5]	unstable
	BBL	-	-4.10[5]	unstable
	LPPV-1	-	-4.50[5]	unstable

*Values obtained from the current study.

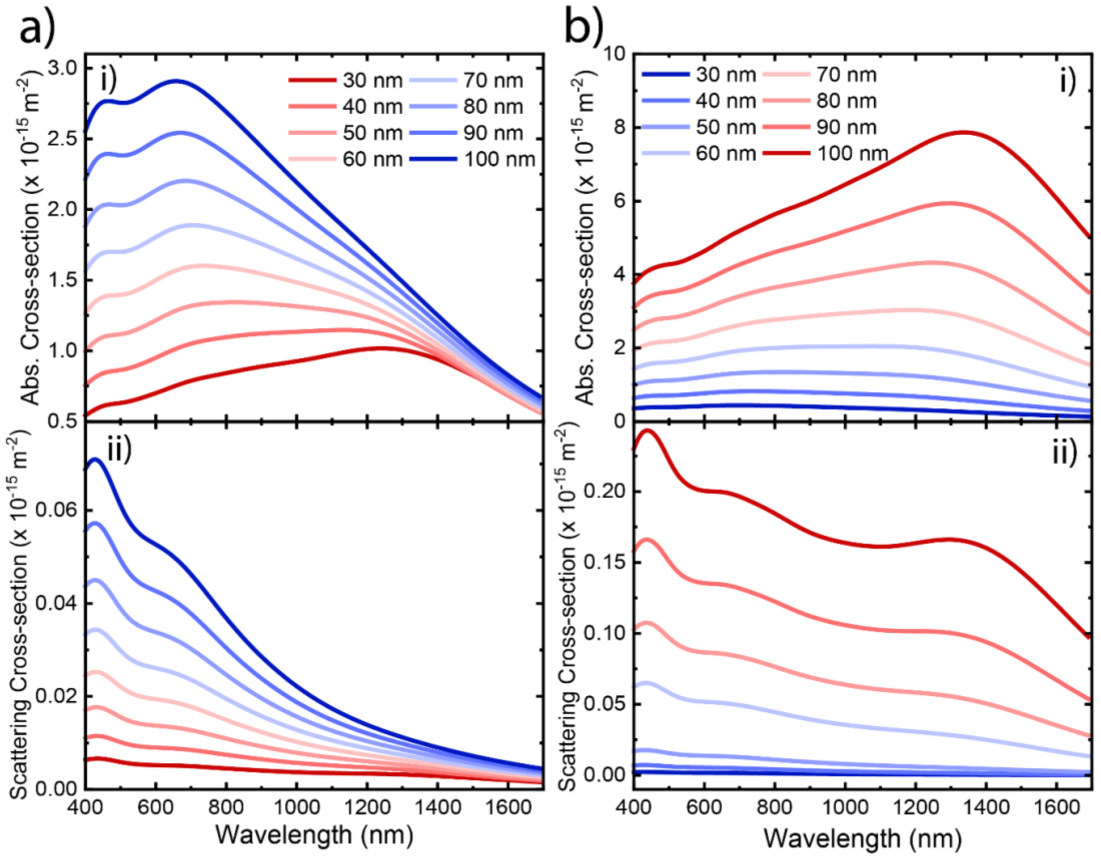

Figure S1: Simulated absorption cross-section (i) and scattering cross-section (ii) of a single nanodisk of PBFDO on glass having a) fixed diameter (50 nm) at varying height (from 30 nm to 100 nm with 10 nm interval), b) fixed height (50 nm) at varying diameter (from 30 nm to 100 nm with 10 nm interval).

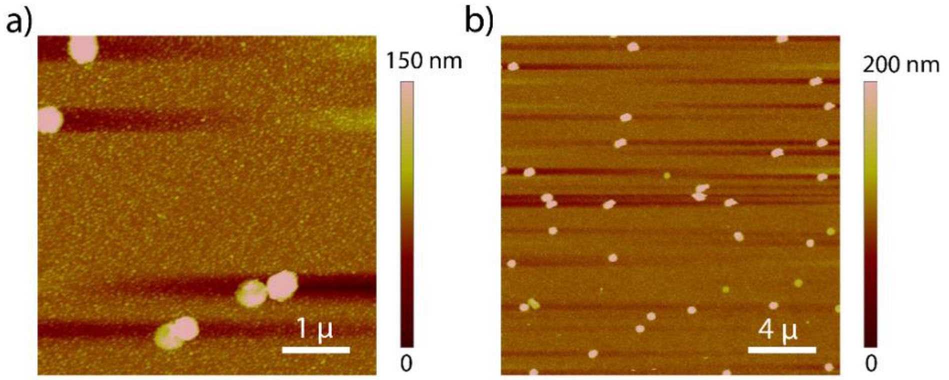

Figure S2: AFM image at scan size of a) 5 µm × 5 µm and b) 20 µm × 20 µm of the PBFDO nanodisks on glass D = 352.9 ± 19.3 nm, H = 113.0 ± 13.8 nm, P = 2073 nm, where D, H and P denotes the average diameter, the average height and the average period. The extinction spectra of this sample can be found in Figure 3a-v.

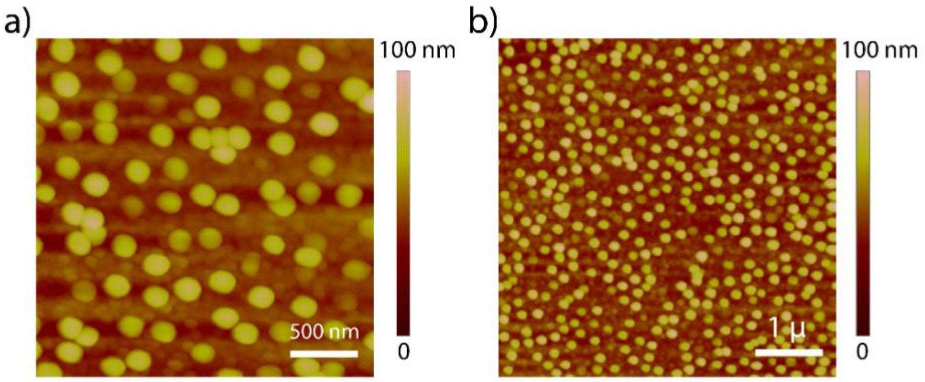

Figure S3: AFM image at scan size of a) 2 µm × 2 µm and b) 5 µm × 5 µm of the PBFDO nanodisks on glass with diameter = 130.9 ± 7.5 nm and height = 35.1 ± 7.3 nm. The extinction spectra of this sample can be found in Figure 4a-ii.

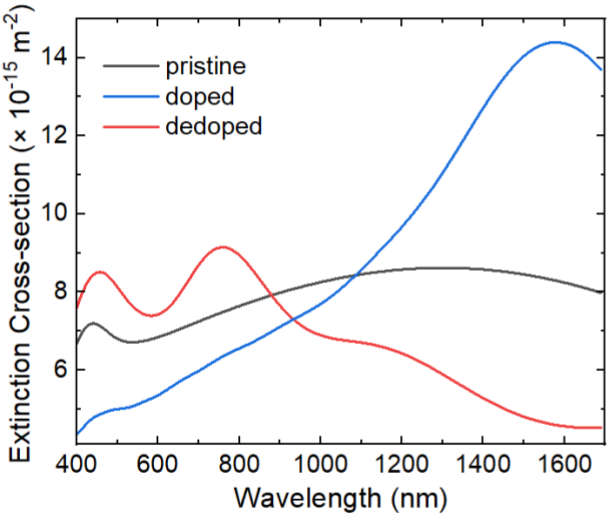

Figure S4: Simulated extinction cross-section of chemically treated PBFDO single nanodisk (131 nm diameter, 35 nm height) in its pristine state (black), PEI-treated doped state (blue) and acid-treated dedoped state (red), corresponding to the experimental results in Figure 4a-ii.

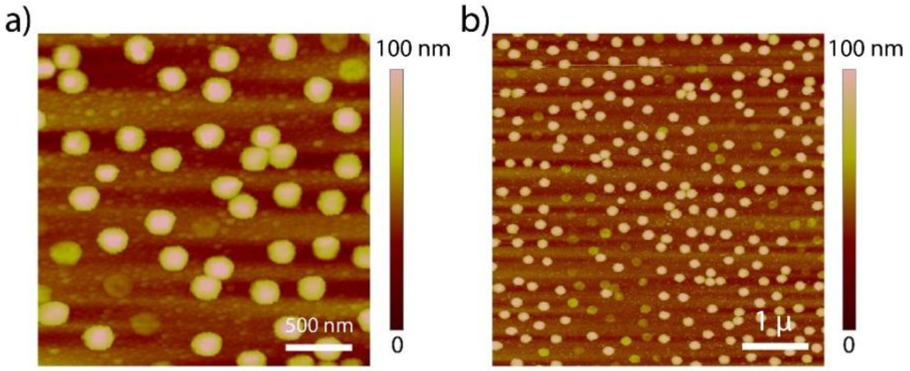

Figure S5: AFM image at scan size of a) 2 µm × 2 µm and b) 5 µm × 5 µm of the PBFDO nanodisks on ITO with 149.3 ± 9.2 nm diameter, 44.3 ± 7.5 nm height. The extinction spectra of this sample can be found in Figure 4b-ii.

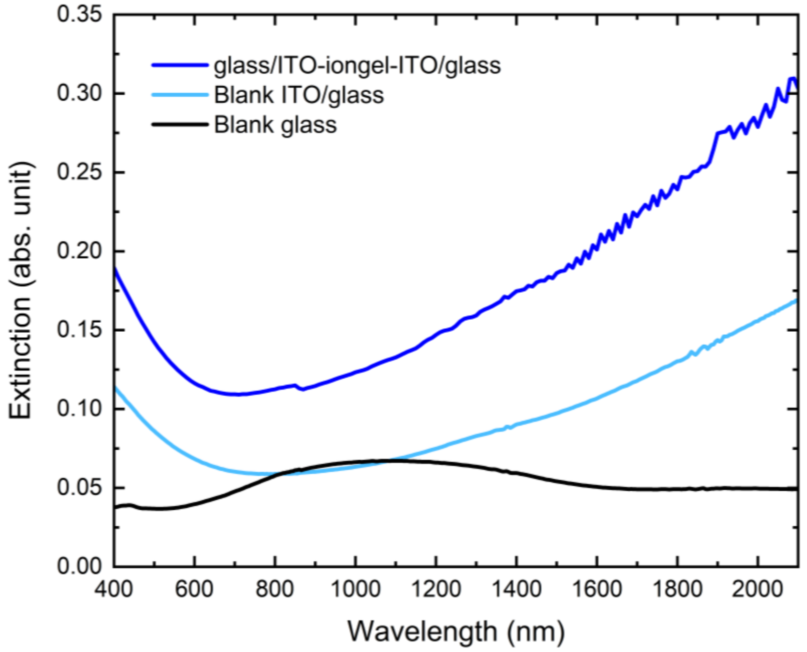

Figure S6: Spectra of different reference sample (blank glass substrate, blank ITO-coated glass substrate and ion gel in between two ITO coated glass substrates) used in this study.

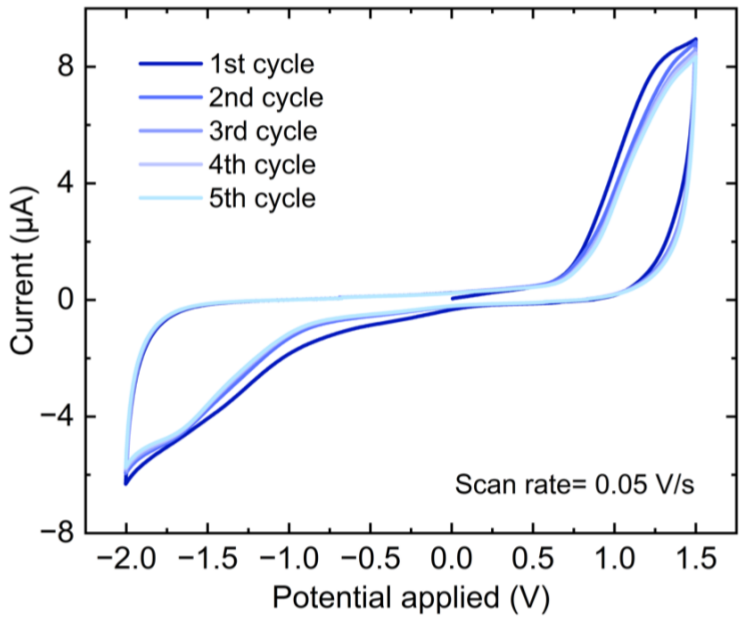

Figure S7: Cyclic voltammogram (CV) of the same PBFDO nanodisk sample as that used for the results in Figure 4b-ii and 4c (diameter = 149 ± 9 nm, height = 44 ± 8 nm) on ITO/glass substrate.

References

1. Hu Q, Yu X, Liu H, Qiu J, Tang W, Liang S, et al. Tunable Organic ENZ Materials with Large Optical Nonlinearity. ACS Photonics 2023;10(10):3612–20.
2. Karst J, Floess M, Ubl M, Dingler C, Malacrida C, Steinle T, et al. Electrically switchable metallic polymer nanoantennas. Science 2021;374(6567):612–6.
3. Chen S, Kühne P, Stanishev V, Knight S, Brooke R, Petsagkourakis I, et al. On the anomalous optical conductivity dispersion of electrically conducting polymers: Ultra-wide spectral range ellipsometry combined with a Drude-Lorentz model. J Mater Chem C 2019;7(15):4350–62.
4. Chen S, Kang ESH, Shiran Chaharsoughi M, Stanishev V, Kühne P, Sun H, et al. Conductive polymer nanoantennas for dynamic organic plasmonics. Nat Nanotechnol 2020;15(1):35–40.
5. Ke Z, Abtahi A, Hwang J, Chen K, Chaudhary J, Song I, et al. Highly Conductive and Solution-Processable n-Doped Transparent Organic Conductor. J Am Chem Soc 2023;145(6):3706–15.
